# Short-Term Memory Trace in Rapidly Adapting Synapses of Inferior Temporal Cortex

**DOI:** 10.1371/journal.pcbi.1000073

**Published:** 2008-05-16

**Authors:** Yasuko Sugase-Miyamoto, Zheng Liu, Matthew C. Wiener, Lance M. Optican, Barry J. Richmond

**Affiliations:** 1 Laboratory of Neuropsychology, National Institute of Mental Health, National Institutes of Health, Department of Health and Human Services, Bethesda, Maryland, United States of America; 2 JSPS Overseas Research Fellow, Japan Society for the Promotion of Science, Tokyo, Japan; 3 Neuroscience Research Institute, National Institute of Advanced Industrial Science and Technology, Tsukuba, Japan; 4 RY84-202, Applied Computer Science and Mathematics Department, Merck Research Laboratories, Rahway, New Jersey, United States of America; 5 Laboratory of Sensorimotor Research, Intramural Research Program, National Eye Institute, National Institutes of Health, Department of Health and Human Services, Bethesda, Maryland, United States of America; University College London, United Kingdom

## Abstract

Visual short-term memory tasks depend upon both the inferior temporal cortex (ITC) and the prefrontal cortex (PFC). Activity in some neurons persists after the first (sample) stimulus is shown. This delay-period activity has been proposed as an important mechanism for working memory. In ITC neurons, intervening (nonmatching) stimuli wipe out the delay-period activity; hence, the role of ITC in memory must depend upon a different mechanism. Here, we look for a possible mechanism by contrasting memory effects in two architectonically different parts of ITC: area TE and the perirhinal cortex. We found that a large proportion (80%) of stimulus-selective neurons in area TE of macaque ITCs exhibit a memory effect during the stimulus interval. During a sequential delayed matching-to-sample task (DMS), the *noise* in the neuronal response to the test image was correlated with the *noise* in the neuronal response to the sample image. Neurons in perirhinal cortex did not show this correlation. These results led us to hypothesize that area TE contributes to short-term memory by acting as a matched filter. When the sample image appears, each TE neuron captures a static copy of its inputs by rapidly adjusting its synaptic weights to match the strength of their individual inputs. Input signals from subsequent images are multiplied by those synaptic weights, thereby computing a measure of the correlation between the past and present inputs. The total activity in area TE is sufficient to quantify the similarity between the two images. This matched filter theory provides an explanation of what is remembered, where the trace is stored, and how comparison is done across time, all without requiring delay period activity. Simulations of a matched filter model match the experimental results, suggesting that area TE neurons store a synaptic memory trace during short-term visual memory.

## Author Summary

To know whether one is looking at an object seen a few seconds ago or not depends on visual short-term memory. To study short-term memory, we recorded single neuronal activity from two brain areas of monkeys, the TE and the perirhinal cortex of the temporal lobe, known to be important in visual pattern recognition and memory. The monkeys performed a short-term visual memory task, a sequential match-to-sample. The monkeys had to signal when a sample stimulus reappeared in a short sequence of stimuli. In area TE only, small fluctuations occurring for a sample-elicited response were correlated with the responses when a match stimulus reappeared, as if a snapshot of the sample-induced response was stored and recalled. In our modeling, we propose that each TE neuron stores and compares the signals during short-term memory by storing the response to the sample in local and rapidly adapting synapses. Subsequent stimulus-elicited responses are then automatically multiplied by the locally stored signal. Here, we show that the match can be detected when the sum of the outputs of the population of TE neurons crosses a threshold. Correlated fluctuations will be a signature this type of local memory storage wherever it occurs in the brain.

## Introduction

Visual short-term, or working, memory is often tested with a sequential delayed match-to-sample (DMS) task. First an image to be remembered (the sample) is presented. Then a sequence of images (the tests), separated by short delays, is presented. The subject is supposed to respond when the remembered image reappears (the match trial). The comparison between images presented at different times requires the brain to compare its current neuronal response with the one that occurred earlier. How this memory task is performed is not well understood, but where it is performed is well known. Analysis of behavior following selective ablations has shown that two large brain regions are important for performing this task: inferior temporal cortex (ITC) and prefrontal cortex (PFC) [1]–[4]. Selective ablations within ITC, particularly perirhinal cortex, interfere with visual memory [5]–[9], but ablations of area TE have different effects than ablations of perirhinal cortex [10],[11]. For example, after area TE ablation, memory at both short and long delays is impaired, whereas after ablations of perirhinal cortex only memory at long delays is impaired [11].

Neurons in both area TE and perirhinal cortex are selective for visual patterns [12]–[15]. In match-to-sample or stimulus-stimulus association tasks, the selective neuronal activity representing the sample or pair-associate image persists during the interstimulus interval for a minority of neurons in both area TE and in perirhinal cortex [16]–[19]. This delay period activity has been thought to play a critical role in maintaining short-term memory. However, the delay-period activity in perirhinal neurons is less consistently selective for the sample stimuli after distractors are presented [15],[20].

Delay period activity during the DMS task is also found in lateral PFC, but in less than half of the neurons [20]. This activity persists and keeps its selectivity for the sample despite distractors [20]. The delay-period activity in prefrontal cortex has also been linked to motor-response selection [20]–[29].

Stimulus-selective delay-period activity has been hypothesized to be the memory trace, and consequently short-term memory has been extensively modeled with attractor networks or feedback networks that maintain their activity after the stimulus goes away [30]–[37]. In contrast, Eskandar et al. [38] developed a multiplicative neural network model that successfully predicted the responses (of ITC neurons in area TE) to both matching and nonmatching test images. In their experimental data few neurons showed stimulus-selective delay-period activity [12]. Thus, their model did not depend on a reverberating circuit, and in fact did not propose any mechanism for storing the memory trace. Instead, it proposed a generic model that used correlation of a stored sample response that was somehow “played back” and compared with each test response. Here we report data from a DMS task showing that single neurons in area TE, but not perirhinal cortex, of inferior temporal cortex, have significant trial-by-trial correlations in the fluctuation of their activity (noise) across sample and match periods. These correlations suggest that some proportion of the neuronal response elicited by the sample stimulus is stored locally, and acts on subsequent stimulus elicited activity.

We present a computational model, based on single-trial learning in a matched filter, showing that the observed correlations could arise from storage of a working memory trace using rapid, short-term synaptic plasticity, and show how the outputs of these neurons could be utilized to detect the match. In this model, the brain does not detect the noise correlations themselves, but simply looks at the total level of activity in the TE neurons to perform the DMS task. Nonetheless, the noise correlations are important because many models of brain function could reproduce the DMS behavior of the monkeys, hence correct performance by itself is not a good criterion for selecting a model. For example, consider recording music on either an analog magnetic tape or on a digital memory stick. If you play back either recording, they will both reproduce the music. And, if you repeat the recordings hundreds of times, the average sound reproduction across these trials will be the same from both. However, if you carefully analyze the sound from each trial, certain systematic anomalies will arise. The analog tape will not move at constant speed, giving rise to shifts in frequencies (wow and flutter), and the digital recording will show only a finite set of levels (quantization). These imperfections have nothing to do with the task of reproducing the sound, but are a unique signature of the recording mechanism and can be used to differentiate between them. Similarly, we argue that the noise correlations are a clue to the mechanism used in short-term visual memory. The matched filter is performing the DMS task by noticing when the overall response is high, but it is also leaving the signature of its mechanism on the responses in the correlated noise. Thus, we can use the correlated noise to infer something about the mechanism, even though it is an epiphenomenon unnecessary for the DMS task.

## Results

**Figure 1:**
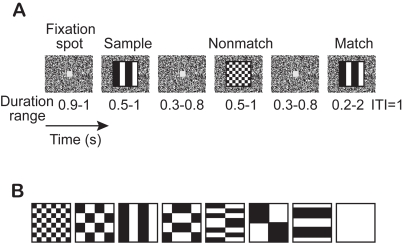
Sequential delayed match-to-sample task. (A) Event sequence. First, a gray fixation spot appears in the center of the screen. Once the monkey fixates on the spot, a sample image replaces the fixation spot for 0.5–1.0 s, after which the spot is restored. After a variable delay-period, the image/spot sequence is repeated 0, 1, 2, or 3 times with nonmatching patterns. Finally, the original (matching) pattern reappears; the monkey has to release the bar within 2 s to get a drop of water as a reward. (ITI, inter-trial interval). (B) Stimuli.

We collected responses from two different parts of ITC: 35 TE neurons and 11 perirhinal neurons from two monkeys performing a visual DMS task (Figure 1A) using eight familiar stimuli (Figure 1B). About 45% of trials had no nonmatch stimuli (sample-match) and about 45% of trials had one nonmatch stimulus (sample-nonmatch-match); the other 10% of trials had two nonmatch stimuli (sample-nonmatch-nonmatch-match) to keep the monkeys attentive to the task. Each picture from the stimulus set was presented as the sample 7–82 times for TE neurons, and 3–82 times for perirhinal neurons. The sample stimulus elicited responses between 0 and 115 spikes/s (median = 10 spikes/s) for TE neurons, and between 0 and 78 spikes/s (median = 10 spikes/s) for perirhinal neurons. In TE, responses were excitatory in 20 neurons, inhibitory in 2 neurons, and either excitatory or inhibitory depending on stimulus pattern in 13 neurons (p<0.05, paired Wilcoxon test). In perirhinal cortex, responses were excitatory in 7 neurons, and were either excitatory or inhibitory depending on stimulus pattern in 4 neurons.

**Figure 2:**
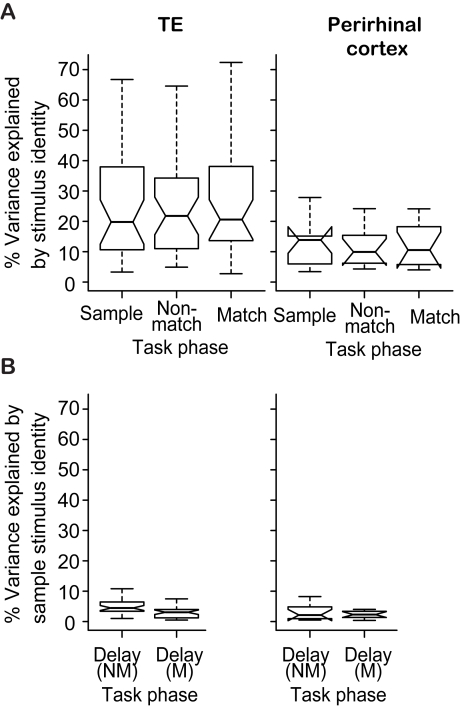
Response variance explained by stimulus identity, demonstrating that these cells were stimulus selective. (A) Percentage of variance explained by stimulus identity in the sample, nonmatch, and match task phases for the population of 35 TE neurons, and for the population of 11 perirhinal cortex neurons. In box plots, the middle line indicates the median. The notches indicate the 95% confidence interval for the median. The whiskers extend to the most extreme data point which is no more than 1.5 times the inter-quartile range from the box. Population distributions across both TE and perirhinal cortex explained the same amount of variance in the sample, nonmatch and match phases. However, in some cells, the behavioral phase significantly influenced the response magnitude. (B) percentage of variance explained by sample stimulus identity in a 200-ms delay period before nonmatch stimulus presentation (Delay(NM)), and before match stimulus presentation (Delay(M)).

The stimulus-elicited responses of both TE and perirhinal neurons were stimulus selective, as expected [12],[13],[39]. In area TE, the effect of the stimulus identity was significant in the sample, nonmatch, and match phases for 29, 34, and 32 of the 35 neurons, respectively (Figure 2A; response variance explained; 1-way ANOVA, p<0.05). In perirhinal cortex, the effect of the stimulus identity was significant in the sample, nonmatch, and match phases for 9, 9, and 8 of the 11 neurons, respectively. Stimulus selectivity explained 26% (mean) of the response variance in TE, and 13% in perirhinal neurons (Figure 2A).

The responses of 16 out of 35 TE neurons had a significant contribution from task phase, i.e. factors indicating sample, nonmatch, and match, (variance explained using 2-way ANOVA with factors “stimulus identity” and “task phase”, R^2^ = 4.1±0.5%, mean±standard error of the mean, p<0.05), consistent with other studies [12],[13]. For 3 out of the 11 perirhinal neurons the contributions of task phase were significant (R^2^ = 3.8±1.7%).

In the inter-stimulus delay periods, for the 35 TE neurons, the contribution of the sample stimulus identity was small but significant for 12 neurons in the delay-period between sample and nonmatch stimulus presentation (a 200-ms period before nonmatch stimulus presentation), and for 2 neurons in the delay period between nonmatch and match stimulus presentation (a 200-ms period before match stimulus presentation) (Figure 2B, left). The effect of the sample stimulus identity was not significant for any of the 11 perirhinal neurons in the two delay-periods (Figure 2B, right). Thus, in our sample of neurons in two parts of IT cortex, area TE, but not perirhinal cortex, had delay-period activity that was (weakly) related to the sample stimulus.

**Figure 3:**
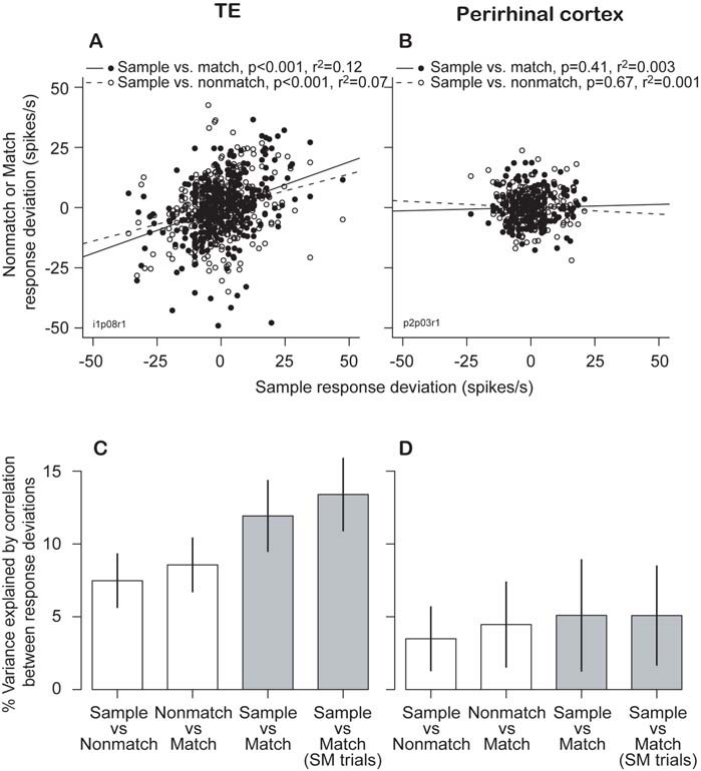
Correlations of response deviations. (A, B) Correlations between sample versus match (filled circles, solid line) and sample versus nonmatch response deviations (open circles, broken line) for one TE (A) and one perirhinal neuron (B). The correlation for sample vs. match is significantly different than for sample versus nonmatch deviations in (A) (Z statistic = 2.11, df = 366, p = 0.018). (C, D) Mean±SE of variance of response deviations explained by TE (C) and perirhinal (D) populations. Differences between sample vs. match and other comparisons (either gray versus either white bar) were significant only in TE (paired t-test, p<0.05).

To quantify the response variation (noise), the phase- and stimulus-dependent mean spike count for each neuron was subtracted from the spike count on each trial in the corresponding task phase. These residuals (noise) were not dependent on the stimulus (1-way ANOVA). However, the deviations during different task phases were correlated with each other, that is, when the response to the sample was above the mean, the response to the match was also likely to be above the mean. On a cell-by-cell basis, the correlations between the sample vs. match deviations were greater than the correlations between the sample vs. nonmatch deviations for most (28/35 = 80%) of the TE neurons (cf. example neuron in Figure 3A). In the sample-nonmatch-match trials, the correlation between deviations for sample and nonmatch images (variance accounted for by linear regression, R^2^ = 7.5±1.8%, N = 35) was weaker than the correlation between deviations in sample-match trials with no intervening nonmatch image (R^2^ = 13.4±2.5%, N = 35; Figure 3C; paired t-test, p<0.05). Thus, for periods separated by the same amount of time, sample-match correlations are stronger than sample-nonmatch correlations.

In sample-nonmatch-match trials, the correlations between sample and match (R^2^ = 11.9±2.4%, N = 35) were still stronger than those between sample and nonmatch, even though the time between sample and match was longer and interrupted by a nonmatch stimulus. Together, these comparisons show that the correlations are not caused by a time-dependent process unrelated to the DMS task. In contrast, the fluctuations in perirhinal neurons (cf. example in Figure 3B) were not significantly different between any conditions (for all pairs: R^2^<4.5%, N = 11, Figure 3D; paired t-test not significant).

For TE neurons, in the sample-nonmatch-match trials, the correlation between the sample response deviations and activity during the delay-period between sample and nonmatch stimulus presentation (R^2^ = 4.3±1.2%, N = 35, significant for 23/35) was weaker than the correlation between deviations for sample and nonmatch images (paired t-test, p<0.01). The correlation between the sample response deviations and activity during the delay-period between nonmatch and match stimulus presentation (R^2^ = 3.4±0.7%, N = 35, significant for 18/35) was weaker than the correlation between deviations for sample and match images (paired t-test, p<0.001). The correlations for these two delay periods against the sample response deviations were not significantly different (paired t-test).

To check whether the observed correlation effects might have arisen by chance, we shuffled the match responses within stimulus pattern group to break the serial relationships between responses within single trials of the DMS task. This shuffling retained the mean response for each stimulus, but broke temporal relations within single trials. All of the response correlations across time fell to nearly zero after shuffling (R^2^ = 0.6±0.1%), and were significantly different from the correlations before shuffling (paired t-test, p<0.001).

Finally, to investigate whether the duration of the delay interval affected the noise correlations, the data were partitioned by delay length. The sample-match noise correlations in sample-match trials with the delays in the range 0.3–0.5 seconds were indistinguishable from those with delays in the range 0.5–0.8 seconds (paired t-test, not significant).

### Model Structure

The experimental results described above lead us to hypothesize that the noise correlation is related to short-term memory, i.e., that the correlated noise is a side-effect of the mechanisms of short-term memory. Our new hypothesis of short-term memory storage and recognition processes is similar to an engineering tool called a matched filter, which is commonly used (e.g., in radar, radiology, etc.) to compare an unknown signal with a known signal [40].

**Figure 4:**
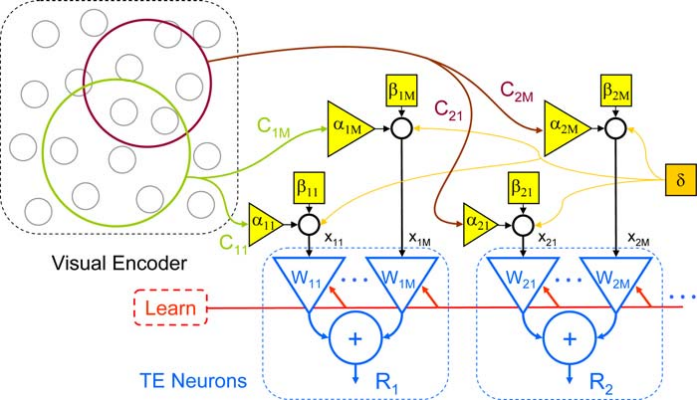
Matched filter model. The matched filter model calculates the correlation between a stored pattern and an incoming pattern. A local memory trace is stored in synaptic weights (*W_km_*) of TE neurons (blue) when a behaviorally determined learning command (red) is sent to the neurons. The image is encoded by a rich array of neurons in the visual encoder (gray circles). The *C_km_* represents the output from a sparse subpopulation of the encoder cells. The green and brown circles represent two overlapping subpopulations that project to different TE neurons. The yellow blocks represent noise between the encoder and the TE neurons; the *α_km_* are independent, multiplicative noise; the *β_km_* are independent additive noise; the gold block (*δ*) represents noise common to all neurons constant throughout one trial (e.g., due to arousal). Thus, on each trial for each stimulus presentation, a new value of *α* and *β* are drawn for each synapse, but only a single value of *δ* is drawn. The *x_km_* represents the input to the synapses, after the noise has been added, and *R_k_* represents the output of the *k*-th TE neuron.

Signal encoding and learning mechanisms are required in any episodic memory model, but we do not speculate about them here. We also deal only with the information processing required, and not with details (architecture, connections, and dynamics) of how a neuronal circuit in cortex could perform the processing. We concentrate instead on the model's memory architecture. When the image is the sample to be remembered, a learning command (*Learn* in Figure 4) causes each input synapse of a TE neuron to set its weight proportional to its current input (the learning mechanism is not specified here, but any short-term process that made the synaptic excitability high after strong inputs and low after weak inputs would be sufficient.) During the sample presentation of the *i*-th pattern, the image is sparsely encoded across all N axons that project to TE neurons. A non-exclusive subset of these N axons then project to a given TE neuron (Figure 4). We model the connections from the encoder population to the TE neuron with algebraic synapses (i.e., inputs are graded, and can be positive or negative). The nature of the encoder is not specified here. It is simply assumed that after the presentation of the *i*-th pattern the spike count on the *m*-th axon branch is *c_mi_*.

Our results deal with two aspects of the data, the average response of the neurons in the DMS task, and the noise on individual responses. To analyze these two aspects, we present the model in two forms, a deterministic model that predicts the average responses of the neurons, and a stochastic model that predicts the noise correlations. In the deterministic model no noise is added to the encoder's activity, so the input to the *m*-th synapse on the *k*-th neuron after the *i*-th pattern is simply:
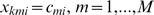
(1)where *M* is the total number of synapses on the *k*-th neuron.

In the stochastic model, which is used to predict the correlated noise seen in our experiments, several types of noise are added to the encoder's output. First, there is a common noise term (δ) that is added to each encoder output, representing a level of arousal. Each encoder also has two independent noise contributions drawn, for each synapse, from a distribution common across synapses: a multiplicative (α) and an additive (β) noise. The input to the *m*-th synapse on the *k*-th neuron is then:
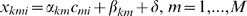
(2)where *α_km_*, *β_km_*, and *δ* are samples of independent noise sources (*α*has mean 1 and *β* and *δ* have mean zero), and *M* is the number of synapses on the downstream neuron. All noise in the stochastic model is referred to the output of the encoder neurons. The samples of noise are drawn each time they are needed (e.g., three times for sample-nonmatch-match trials). The *c_mi_* thus represent the average, or expected value of each encoder output for a given stimulus, and the *x_kmi_* represent the particular (noiseless or noisy) sample.

### The Synaptic Weight Memory Trace

When no learning is present in the model (e.g., for perirhinal neurons), the synaptic weights at the *m*-th synapse of the *k*-th neuron are all set to unity gain:
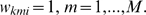
(3)


When the *i*-th pattern is shown and learning is triggered, the weight (strength) of the *m*-th synapse of the *k*-th neuron receiving that input is set to:
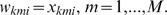
(4)These synaptic weights are the memory trace. For each subsequent image the output of the TE neuron will be the product of the encoder output elicited by the test stimulus and the synaptic weights that hold the memory trace. Thus, the output of each TE or perirhinal neuron is a correlation: the sum of the product of the input activity with the stored weights. The response of the *k*-th neuron to the pattern pair (sample *i*, test *j*) is simply the sum over all *M* synapses:
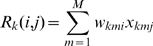
(5)


In the matched filter theory, the test image is considered as a match if the activity summed across neurons is above a threshold. Some subset of neurons will respond strongly (on average) to a given sample pattern. When a nonmatch stimulus is later presented, not all of these neurons will fire strongly, so the product of weights and inputs (Equation 5) for at least some of those neurons will be low (even though the weight is high), and the sum of all those responses will not exceed the threshold for recognizing a match. When a match stimulus is presented, each neuron in the subset will again fire strongly, and be multiplied by the high weight, giving a sum of responses across neurons that exceed the threshold for recognizing a match. Thus, to recognize matches, the matched filter relies on the fact that a neuron that responded strongly to a given stimulus once will, on average, respond strongly to that stimulus again. The matched filter simply relies on standard stimulus selectivity–different stimuli evoke different average responses.

The matched filter mechanism for detecting matches does not make use of, but does give rise to, the “noise” correlations observed in TE neurons in our data. As just explained, the matched filter relies on differences in the average response to different stimuli to detect a match. But the weight stored after presentation of the sample image depends not on the average response to the sample image, but on the individual response to that presentation. The response of a neuron to an individual presentation of the sample image can be higher or lower than the average across presentations for that image. If, on a given trial, the response of a TE neuron to the sample image is higher than average, the synaptic weight will be set higher than the average during that trial. When the match is presented, the encoder signal can be lower, higher, or the same as the average. However, the multiplicative interaction between the input and the weight will bias the average of many such interactions to be higher than average if the stored weight is higher than average. Similarly, lower sample responses lead to lower synaptic weights and a lower than average response to the match. Put another way, the deviations of the responses from their means for the sample and test patterns will be correlated within a trial. Note that whereas individual neurons can be above or below average for any given trial, these fluctuations will average out, and the total activity across the population will only be above threshold for the repeat of the sample image.

We test this model in two ways. First, a single-synapse (scalar) version of the deterministic model was applied to the average data collected in the experiment, to test the model's ability to perform the DMS task, on average, like the neurons. Tests with the average responses can not evaluate the correlation of the response noise across conditions, so a second test was needed. We used a stochastic vector model (shown in Figure 4) with a simple visual encoder and two types of noise (additive and multiplicative) on the input to the downstream neuron's synapses. We also added a noise source shared by all synapses, to test the hypothesis that changes in arousal could explain the noise correlations in our TE data. The model was simulated and the noise parameters were adjusted to best fit the correlation between the noise in the responses to the sample and the noise in the responses to the test stimuli found in the experimental data. It is important to emphasize that this tuning only sets the relative size of the correlations; the fact that there is a correlation depends upon the matched-filter model's structure. Indeed, the same tuning did not create correlations in the stochastic model of the perirhinal neurons, because they have no memory trace.

### Deterministic Model Simulation

The inputs to the model are unknown and must be estimated. It is possible to train a neural network to find the *x_ki_* that solves Equation 5 (not shown). However, this approach yields a model with a very large number of free parameters, and thus provides only weak support for our hypothesis. We can make a stronger test of our hypothesis by noting that in the DMS task there is a special case, the response of a neuron to the matching image, which has the response:
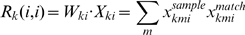
(6)


To compare our model to the data, we can only consider scalar variables, because the single-unit recordings give only the spike count in the response to a stimulus. The individual encoder outputs are not known. Thus, a scalar approximation of the encoder output, *e_ki_*, the unknown input to the *k*-th neuron for the *i*-th pattern, can be estimated as the square root of the average of the responses across *N* repetitions of the match stimulus:
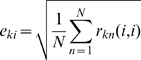
(7)where *r_kn_* is the spike count on the *k*-th neuron on the *n*-th experimental trial. This corresponds to a model where each neuron has only a single synapse. This is obviously a major simplification of the model, but it is necessary because we can not observe the output of the encoder by recording from single neurons. Furthermore, Equation 7 is only a rough approximation, because it takes the square root of the sum, instead of the sum of the square roots, of the individual *r_kn_*. The advantage of this approach, which offsets the coarseness of the approximation, is that we have a *parameter-free, deterministic model that performs the DMS task*, with all of the assumptions explicit in the structure of the matched filter.

The predicted response, r^*^, of a neuron for the nonmatching *j*-th pattern following the sample *i*-th pattern with a memory effect would then be:
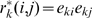
(8)


Similarly, the predicted response of a neuron to the matching *i*-th stimulus would be:
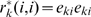
(9)


For completeness, the predicted response of the matched filter model to the sample *j*-th image is calculated based on the assumption that the memory trace is still set to the previous sample, say the *p*-th image (i.e., this assumes the previously stored signal persists until a new learning command occurs). The old memory trace probably decays away over time, but this assumption lets us calculate a conservative estimate of the sample response:
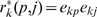
(10)


**Figure 5:**
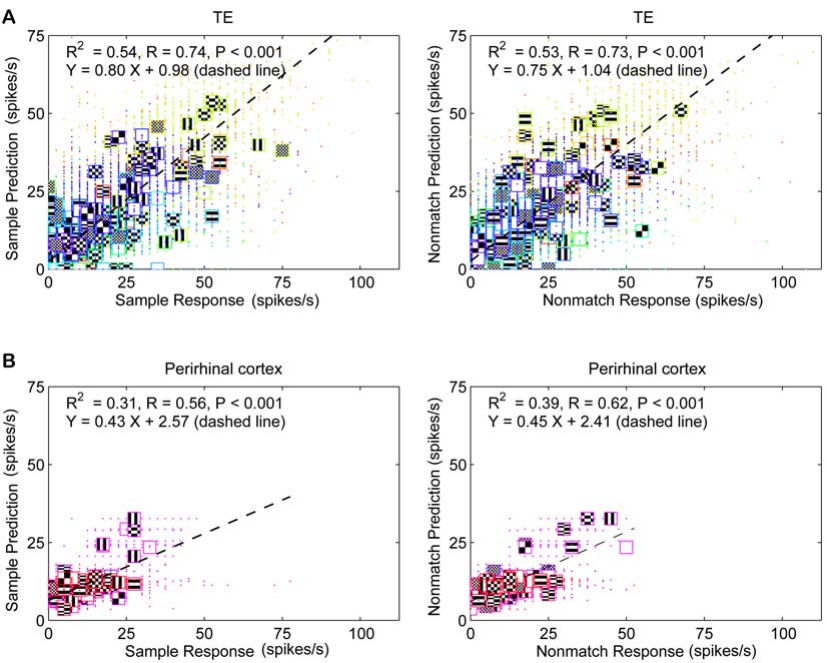
Predictions of responses by the deterministic model simulation. (A, B) Predictions of responses for TE neurons with inputs from encoder stage estimates (Equations 1–10) (A), and by the model for perirhinal neurons (Equation 3 was applied) (B). Left column shows predictions of sample responses compared to the actual sample responses. Right column shows predictions for nonmatch responses. Each colored dot represents data points for each neuron, and each pattern with a colored outline indicates the mean response versus mean predicted response to the pattern for the neuron. Variance explained is high in all cases, because the cells in both areas are stimulus selective.

The encoder output is estimated from the response of the neuron to the match stimulus (Equation 7). Thus, the deterministic model can only be used to predict the responses to sample and nonmatch stimuli (Figure 5). For the population of TE neurons, the correlations between sample response and prediction (R = 0.74) and nonmatch response and prediction (R = 0.73) are significant (Figure 5A; p<0.001; *R^2^* = 0.54 for TE sample predictions, and 0.53 for TE nonmatch predictions). For the population of perirhinal neurons, the correlations are lower, but still significant (Figure 5B; p<0.001, R^2^ = .31 and 0.39, sample and nonmatch, respectively). This is consistent with our expectations, because both types of neurons showed stimulus selectivity (see Figure 2A). Thus, the scalar matched filter model, with no free parameters and with the simplistic approximation of Equation 7, successfully predicts the responses of the neurons during the DMS task, accounting for a bit more than 50% of the variance in the TE data.

**Figure 6:**
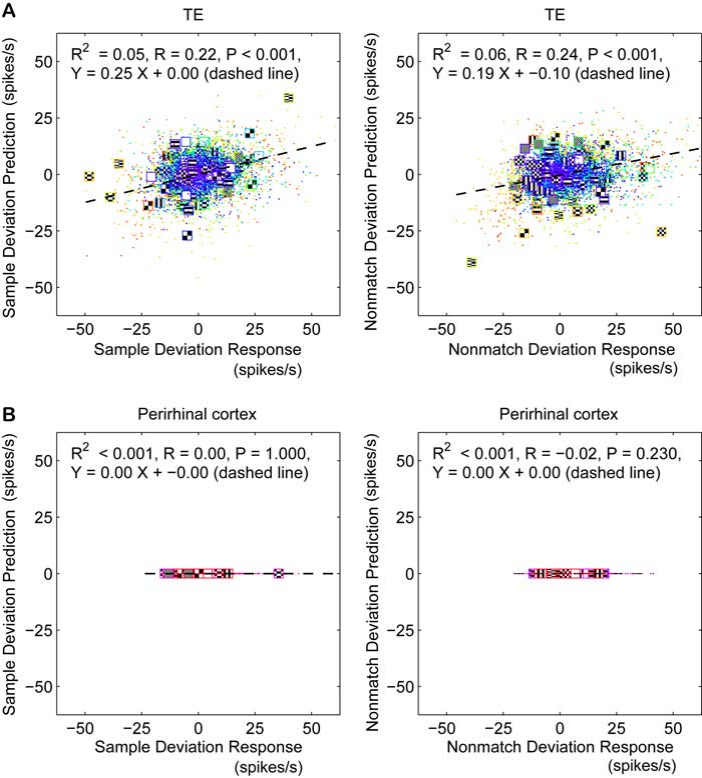
Predictions of response deviations by the deterministic model simulation. (A, B) Predictions of the deviations for TE (A) and perirhinal (B) neurons. Left column shows predictions of the deviations from the mean in the sample responses compared to the actual sample response deviations. Right column shows predictions for nonmatch response deviations. Variance explained is low in TE, but zero for perirhinal, which is a rough match to our data. (Format as in Figure 5.)

In Figure 6, instead of predicting the responses of the neurons to the sample or nonmatch stimulus on each trial, we predict the deviation of that response from its mean. This roughly matches what we found in the data (see Figure 3). The variance explained in the prediction of the response deviation was much less than the variance explained in the prediction of the response itself for perirhinal neurons (R^2^ = 0.05 and 0.06 for sample and nonmatch deviations in TE, and R^2^<0.001 for deviations in perirhinal neurons).

### Implications for Population Coding

**Figure 7:**
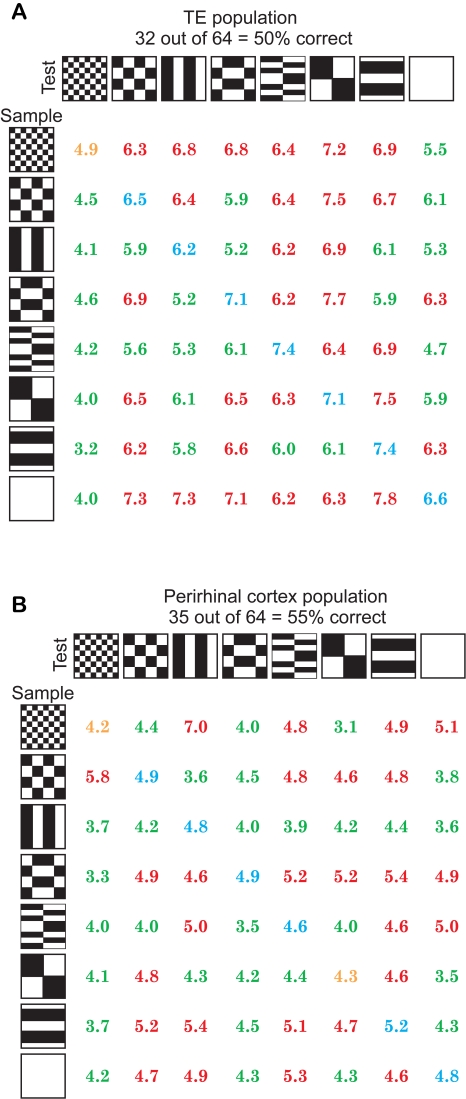
Performance on the DMS task of the deterministic matched filter model. (A) Performance of the deterministic matched filter model using the data from the recorded sample of 35 TE neurons. The left column shows the eight stimuli presented to the model as the sample, and the top row shows the eight stimuli presented as the test image. At the intersection of each row and column is the average response of all the estimates across 35 TE neurons using the matched filter model. The upper-left to lower-right diagonal shows the matched filter outputs for the eight sample-match pairs. The off diagonals show the matched filter outputs for the 56 sample-nonmatch pairs. The model gave the best discrimination performance with the threshold set to 6.15 spikes per 400 ms epoch, i.e., the model made the fewest mistakes. The blue values show correct matches (hits), and the green responses show the correct nonmatches (correct rejections). The orange values show misses, and the red values show false alarms. This model got 32/64 = 50% of the trials correct. (B) Performance of the matched filter model for perirhinal neurons with inputs from encoder stage estimate. At the intersection of each row and column is the average response of all the estimates across 11 perirhinal neurons using the matched filter model. With the threshold set to 4.55 spikes per 400 ms epoch, the model achieved its best performance, getting 35/64 = 55% of the trials correct.

Figure 7A shows the results of computing the match-nonmatch performance for the set of 64 population responses for the 35 TE neurons. Each row (sample) and column (test) begins with the corresponding stimulus. The average population response is printed for each nonmatch and match decision. The diagonal values show the match responses (in spikes per 400 ms epoch). A number colored in blue is a correct match decision (or hit, based on a threshold of 6.15), and an orange number is a miss. The off-diagonal elements show the nonmatch responses. A green number is a correct rejection, and a red number is a false alarm. Overall, the matched filter based on these 35 neurons scored 50% correct (ROC d' = 1.02; random would have been 1/64 or 1.56% correct) on the DMS task. The same comparison is made in Figure 7B for the perirhinal neurons, which scored 55% correct (d' = 0.72). The similarity in scores is not surprising, as TE neurons project to perirhinal cortex. However, the d' value (which is the separation of the means of the probability density functions of occurrence, with and without signal, divided by the standard deviation of the distributions) is much smaller in perirhinal neurons. This suggests that signals that were separate in TE have become confounded in perirhinal cortex.

### Stochastic Model Simulation

The second test of our model is whether it can give rise to noise correlations, for which the noise on the visual encoder must be modeled parametrically. In the stochastic vector model (Equation 2), there are three parameters (to specify the variances of the three noise processes α, β and δ). Note that these three parameters are fit independently in the model for both TE and perirhinal neurons (see Methods), but their presence alone is not sufficient to generate the noise correlations in our data. It is the presence of learning that introduces the noise correlations, which is clear because the perirhinal neurons do not learn, and do not show this correlation.

**Figure 8:**
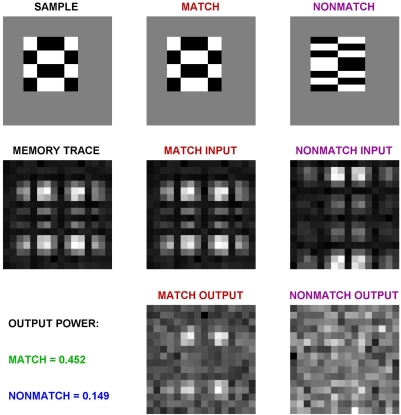
Example of matched filter output computation. The top row shows input images. The memory trace of the model is simulated with the discrete Fourier Transform of the input, plus noise (middle row). (Noise is not very noticeable, because of the logarithmic scaling). Bottom row shows the product of the memory trace and the match and nonmatch inputs. The output power is shown on the left.

Above, predicted responses were computed from the average responses of the experimental data. To simulate the DMS task with a matched filter model with noise on a trial-by-trial basis, we need to generate an encoder output. For simplicity, we chose the discrete Fourier transform (DFT) to represent the encoder. Each 8×8 stimulus was placed on a 16×16 gray background. The stimuli (Figure 8, top row) were first converted to their 16×16 DFTs (Figure 8, middle row). Each DFT image thus represents activity in 256 encoder cells (represented as a vector of length 256). The output of the model is just the dot product of the sample and test responses (Figure 8, bottom row. NB: the luminance levels in the figure are a poor indicator of their importance, because of the log transformation used in plotting). The average output power (calculated using root-sum-of-squares of population activity, with the brightest pixel across all stimulus pairs normalized to 1.0) across the entire population is given on the left (0.452 for the match, and 0.149 for the nonmatch case).

**Figure 9:**
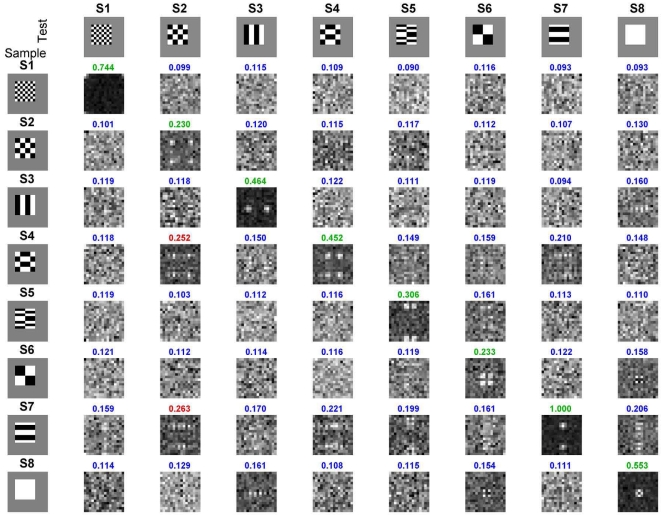
Performance of the matched filter across full set of stimulus pairs by the stochastic model simulation. The left column shows the 8 stimuli presented to the model as the sample, and the top row shows the 8 stimuli presented as a match or nonmatch. The intersection of each row and column is a 16×16 pixel image made up of the responses of the 256 model TE neurons. The diagonal (with slope -1) shows the matched filter outputs for the eight sample-match pairs. The off diagonals show the matched filter outputs for the 56 sample-nonmatch pairs. The total power (normalized to 1.0 for the peak of the 64 pair set, in this example, S7-S7 sample-match) is shown above each output. With the threshold set to 0.225, the model made the fewest mistakes (false alarms, red values). The green values show correct matches, and the blue responses show the correct nonmatches. With the noise in the model adjusted to match that in the monkeys, the model got 62/64 = 97% of the trials correct. The average performance across the two monkeys was 98%.

The output results for all 64 combinations of stimulus and test patterns are shown in Figure 9. As in Figure 7, the matches are on the diagonal, and the nonmatches are on the off-diagonals. The normalized output power is printed above each response image for a population of 256 encoder neurons (shown as a 16×16 icon). Green numbers are correct hits, blue are correct rejections, orange are misses (none in this example), and red are false alarms. With the threshold set to 0.225, the model makes only two mistakes (both false alarms). This gives the model a success rate of 97% (d' = 3.34; the correction for *p*(*hit*) = 1 was made using *p*(*hit*) = 1−0.5/(*N_hit_*+*N_miss_*), [41]). The average success rate of our two monkeys was 98%. (These two rates are so close because the noise in the model was tuned to match these monkeys, so it is a fit, not a prediction; see Methods).

**Figure 10:**
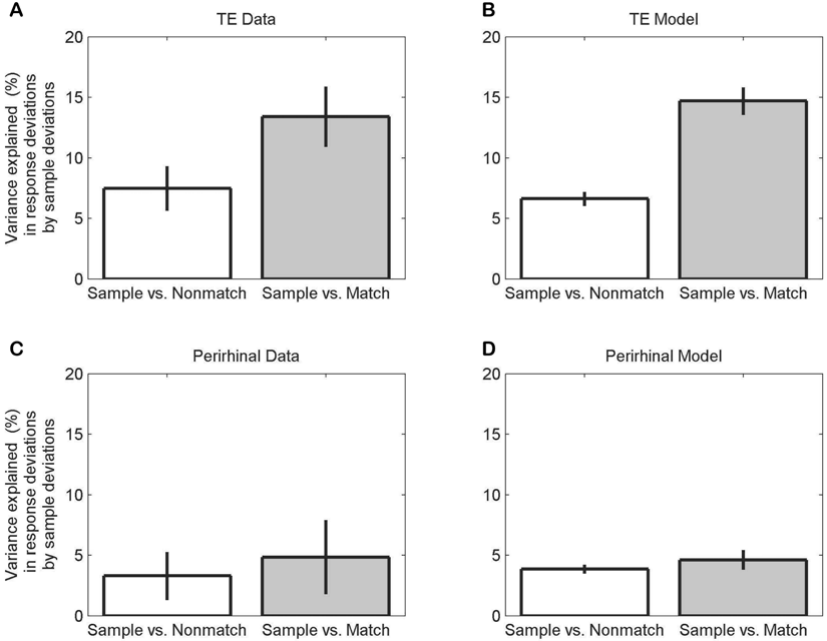
Stochastic model's noise correlations. Correlations for noise in sample versus match and sample versus nonmatch deviations are similar to our data. (A, B) TE shows significantly higher noise correlations for sample versus match phases. (C, D) response deviations in the perirhinal cortex are much less, and more uniformly, correlated. Data are from trials with no intervening nonmatch stimuli for sample versus match or from trials with one nonmatch stimulus for sample versus nonmatch. Uniform correlations in perirhinal cortex are due to slowly varying input noise. Increased sample versus match noise correlations depend on a multiplicative interaction between memory trace and current input.

A quantitative comparison of the performance of the model with that of the monkeys is shown in Figure 10. The average correlations between the sample and match response deviations are shown for actual and simulated TE neuronal responses (Figure 10A and 10B, respectively). The response correlation was larger between the sample and match phase than between the sample and nonmatch phase (for simulated response: paired t-test, p≤0.00001; for actual response: p< = 0.001). As a control, the same model was used to simulate a population of neurons in perirhinal cortex by fixing the synaptic weights (Figure 4, W_km_) to 1.0. Without synaptic plasticity, noise correlations in the perirhinal simulations were the same for all phase pairs (Figure 10D), as was found in the experimental data (Figure 10C). Thus, our simple matched filter model shows that the unexpected correlation between noises at different times for sample vs. match responses is an emergent property of a multiplicative matched filter model that stores its memory trace locally with one-trial learning of synaptic weights.

Note that one parameter in the noise model, δ, is shared by all the TE neurons. It represents a kind of alertness level. If δ varied slowly over time, it could introduce a correlation between sample and test responses. However, as the nonmatch response is always equal to or closer in time to the sample response than is the match response, the effect of the δ noise must make the noise correlations on sample-nonmatch responses the same or larger than the noise correlations on the sample-match response. This is the opposite of our data (sample-match noise correlations were larger, Figure 3). Hence, in this model the δ noise process contributes to the height of the correlations in Figure 10B and 10D, but not to the height differences in either panel. The magnitude of the correlation is also adjusted by tuning the α- and β-noise processes. However, without the multiplicative effects of the matched-filter model there would be no difference in heights of the bars for area TE (Figure 10B). They would be like the bars for perirhinal cortex (Figure 10D).

### Delay Period Activity

**Figure 11:**
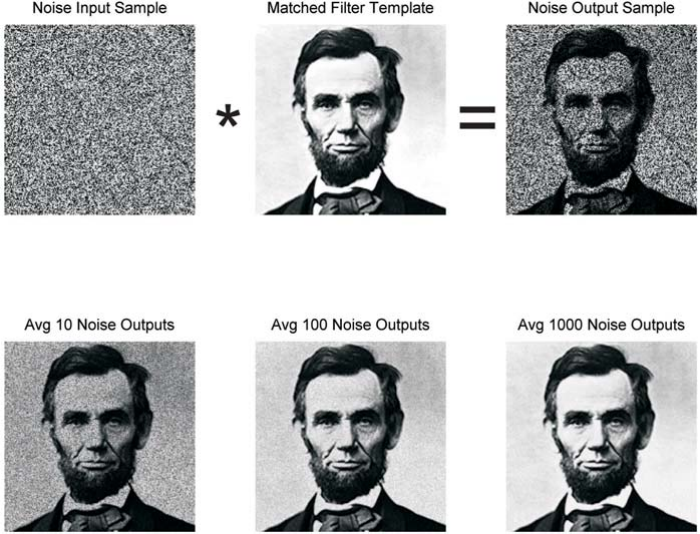
Noise-driven output of a matched filter. Top row shows a sample image made up of white noise uniformly distributed on [0, 1], the template, and the product of the two. The second row shows the effect of averaging across many such representations. Clearly, the signal-to-noise ratio is rapidly improving as the size of the pool being averaged increases, but even the output from a single sample (top row) looks somewhat like the filter. The response to the noise input has revealed some cells with selectivity for the template image. Similarly, neuronal activity seen between stimulus presentations may be the result of noisy inputs to matched filter cells.

The usual interpretation of the average delay period activity in a DMS task is that it reflects activity in a reverberatory circuit (attractor network) that is holding the memory. It is interesting to ask what happens to a matched filter between stimulus presentations. If all inputs are set to zero, then there is no output from the matched filter. However, if there is noise on the inputs to the matched filter during the interstimulus interval, the matched filter would produce an output (Figure 11, top row). The output looks like the template, but with a much reduced signal-to-noise ratio (SNR). If this response to noise were averaged over several trials, the SNR would improve (as the 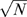, where N is the number of trials in the average; see Figure 11, bottom row). This example shows that another interpretation of the delay-period activity is possible: it may be the response of a matched filter to noise, which reflects the current setting of the synaptic weights.

## Discussion

**Figure 12:**
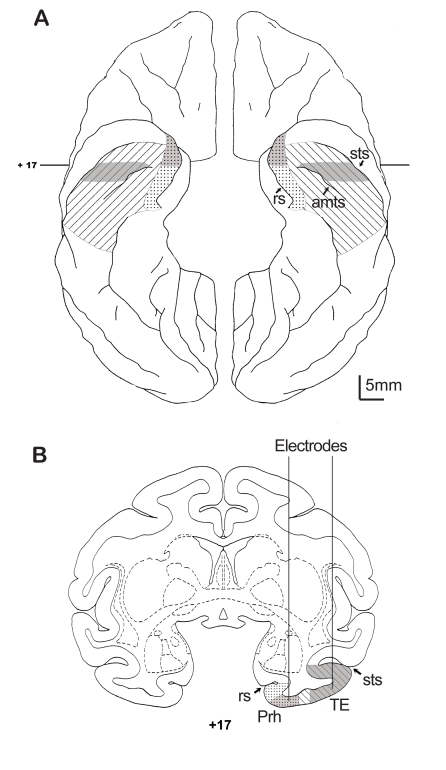
Schematic localization of recording sites. (A) Ventral view of the brain with perirhinal cortex (medial, dots) and area TE (hatched) highlighted. Actual recording was done in parts of area TE and perirhinal cortex that are indicated in gray. (B) Coronal cross-section of a standard rhesus monkey atlas (Laboratory of Neuropsychology, NIMH; http://ln.nimh.nih.gov/) at 17 mm rostral to the interaural line (AP +17) showing a recording track into perirhinal cortex and a track into area TE. The noise correlation was found to occur for most area TE neurons that were recorded lateral to the anterior middle temporal sulcus. The noise correlation was not found in the responses of neurons recorded in perirhinal cortex in this or pervious studies recorded medial to anterior middle temporal sulcus [19],[45]. MRs with electrodes can be seen in Liu and Richmond [13]. amts, anterior middle temporal sulcus; rs, rhinal sulcus; sts, superior temporal sulcus; TE, area TE; Prh, perirhinal cortex.

As information has been collected about localization of memory functions in the brain over the past decade, it has become clear that different architectonic regions of inferior temporal cortex have different functional roles in memory, specifically the lateral inferior temporal area TE has different roles in memory than the more medial inferior temporal perirhinal cortex [10],[11],[42],[43]. Among other differences bilateral ablation of the more lateral area TE interferes with memory at all delays whereas damage to the more medial perirhinal cortex interferes with memory only after longer delays in monkeys [11]. This suggests that area TE is involved in the initial encoding of information for memory formation in general. Relevant to the present study, neurons in both areas show stimulus selectivity, and activity related to stimulus-stimulus associations [43]. However, the stimulus-stimulus association related activity in TE is dependent on perirhinal cortex [44]. Latencies of the visual stimulus elicited responses are considerably shorter in area TE than in perirhinal cortex, and stimulus-elicited reward schedule related selectivity arising from associative learning is seen in perirhinal neurons, but not in area TE neurons [13]. Here, we found that the stimulus-elicited responses recorded between anterior middle temporal sulcus and superior temporal sulcus, area TE (Figure 12), have a short-term memory related signal, and that neurons recorded medial to anterior middle temporal sulcus and lateral to rhinal sulcus, perihinal cortex and perhaps medial area TE, do not have this signal. Our findings in perirhinal cortex are consistent with previous findings in perirhinal cortex [19],[45].

Our recordings from TE neurons during a sequential delayed match-to-sample task show that trial-by-trial neuronal response variability (i.e., noise) is better correlated between sample and match than between sample and nonmatch responses. This can not be because of exogenous factors (e.g., slowly varying arousal) introducing correlated noise into the responses, because the noise was also better correlated between sample and match responses even when there was an intervening nonmatch stimulus, which thus increased the temporal separation of the sample and match stimuli. This suggested that the individual response to the sample picture, and not some average response, was being stored somehow and then recalled at the time that the match stimulus was presented. To interpret this finding, we hypothesize a synaptic storage and recall mechanism: the memory trace of the response to the sample image is held in rapidly adapting weights on the synapses of each TE neuron. TE then acts as a matched filter to compare the new signal with the old one. In a matched filter model, a new picture is broken into pieces by the encoder and distributed across a set of multiply-accumulator elements. Each piece is multiplied by a weight that was set when the sample stimulus was presented. These results are added and compared to a threshold [40]. This converts the time-domain operation of correlating two pictures shown sequentially (referred to as signals) into a spatial operation on the second signal, with the first signal being spatially distributed in the filter's weights.

### Exploiting an Epiphenomenon

The noise correlations arise because of the multiplication stage in the matched filter. The brain cannot detect these noise correlations; it only detects the total activity in the population after the test image is presented. The experimenter can observe the noise correlations in the data, and infer from them something about the mechanism that is acting. That these correlations are irrelevant to performing the task is obvious because of the large number of neurons involved. To make a match or nonmatch judgment, the brain must take some kind of average over a population of neurons. Furthermore, this population must contain about the same number of neurons responding above and below their average responses. Thus, over the population the correlated noise would average out.

Another inference follows from our observations: that the synaptic weights holding the memory trace must be on the TE neurons from which we are recording. The responses of TE neurons can not simply be reflecting an input from a different area, say prefrontal cortex, which was recalling the previous input. If another area held the memory, then they would have to be holding the previous output of area TE. The exact same cells that projected to each neuron in the memory area would have to receive a return projection from that neuron. In other words, the mapping from TE to the memory area would have to be 1:1. No cortical brain area we know of contains a 1:1 mapping. Instead, neurons seem to have a large degree of fan-out and fan-in, so each neuron connects with many others, and many others connect to it. In such a many:many mapping the exact value of the response of the TE neurons to the sample stimulus would be averaged out by the time it returned to TE during recall. But that would destroy the very noise correlations we observed. Thus, the synapses that hold the memory trace must be on the TE neurons themselves.

In our model (Figure 4, and Materials and Methods), the “signals” are the neurons that provide inputs to TE, and the weights are encoded by the strength of their synapses onto TE neurons. Each neuron remembers only its own input, and thus learning happens locally, by the modification of synaptic weights. The model is biologically plausible–the multiplication, addition, and threshold operations are easily available to neurons [46]–and no signals need to be transmitted to, or recalled from, any other part of the brain for comparison. Note that this model has only an implicit recall; there is no actual reconstruction of the original signal to compare with the current signal. However, one surprising feature of the matched filter is that when excited by a random signal (e.g., white noise inputs), the average of its response will be an approximation of the original signal (cf. Figure 11).

Our model is similar to most others formulated to describe memory in that it uses synaptic plasticity to create a stored memory. Here we specifically propose using rapid synaptic plasticity gated by a learning command. Although this rapid type of synaptic plasticity has not been observed, its existence has been hypothesized by others when considering how working memory might arise [47],[48]. Our hypothesis only requires that synapses of TE neurons are altered according to their current input: if a particular input is high the weight is set high and if a particular input is low the weight is set low. The synapse therefore has a memory of the input, which consists of both signal (mean response) and noise (deviation from the mean). Every subsequent signal is multiplied by this adapted synaptic weight. This multiplication correlates the response with the remembered signal–the mean plus the deviation.

### Delay-Period Activity

Our hypothesis of memory storage does not require delay activity, which has been seen mainly in perirhinal cortex [15] and, even there, only a relatively small proportion of neurons show this property [16],[18],[19],[49]. In our data, the noise correlation is significant for most TE neurons, suggesting that most of these neurons participate in this simple working memory. A consequence of the matched filter model (Equation 5) is that the average response over the population shows match enhancement and nonmatch suppression. This is the basis for the filter's discrimination. For an individual member of that population, however, nonmatch responses can be larger than match responses, depending upon the selectivity of the neuron. This can be seen in the simulation by comparing individual pixels (i.e., simulated neurons) down a column in Figure 9.

### Systematic Enhancement or Suppression

A systematic match enhancement or suppression has been seen in neurons in perirhinal cortex (see Figure 12) [14],[15],[49], a cortical region that seems heavily involved in decisions about remembered stimuli [7],[8],[10],[13],[50]. Our data do not show such systematic changes in perirhinal cortex, so our model does not deal with this behavior.

Our model also does not try to account for other effects of novelty, recency or familiarity, in which the responses to previously seen stimuli are sometimes smaller on subsequent presentations, because this is an effect observed mainly in neurons medial to the anterior middle temporal sulcus, in perirhinal cortex [51]. Some cells in perirhinal cortex showed both match suppression (a short term memory effect that decreased responses to the matching stimulus) and a familiarity effect (a long term response decrement) over long times when the stimuli were repeatedly presented [52]. The neurons we recorded that showed the noise-correlation effect were all in area TE, lateral to the anterior middle temporal sulcus, where previous reports did not find match-suppression [12],[49]. The neurons we recorded that did not show the noise-correlation effect were medial to this sulcus, in perirhinal cortex.

### Population Activity, Not Noise, Is Used to Detect the Match

It is important to emphasize that in this model the brain does not perform the DMS task by detecting the noise correlations. The DMS task is performed by comparing the level of activity in a population of TE neurons with a threshold. The threshold determines the sensitivity of the detector, and thus is probably under behavioral control. The noise correlations are observable only by the experimenter after the task, and not by the brain during the task. The noise correlations are thus a clue to the mechanism used to solve the DMS task, in this case, a multiplicative model.

Our findings and model here extend our previous work [12],[38]. In those studies we found that “responses to the nonmatch stimuli carried significant amounts of information about the pattern of the previous sample stimuli.” This is consistent with our findings here, because the response of an inferior temporal (IT) neuron would be the product of the visual codes for the sample and test stimuli. We hypothesized then that “the role of IT neurons in visual memory tasks is to compare the internal representations of current visual images with the internal representations of recalled images.” This is exactly what we are proposing here, but now we have a specific hypothesis for the memory mechanism that eliminates the need for recalling the response to the sample image.

### Other Types of Memory

This new theory is applicable in any area of the brain that depends upon synaptic changes, rather than persistent activity, to hold a memory trace. Other types of working memory should be studied with this in mind. This work may even be relevant in areas that hold a memory as delay-period activity in an attractor network, such as prefrontal cortex, because synaptic plasticity is required to create the attractor representing the object that is being remembered [31],[37].

There are many forms of memory, and DMS just tests one particular type of explicit, or declarative, memory. For example, Standing [53] studied free-recall or recognition tasks. He showed that thousands of pictures or words could be recognized as familiar after being seen only once. As Standing pointed out, this is different from the limited “memory-span” (about seven items) required to deal with ordered lists. Although the DMS task is more like a memory-span task than a familiarity task, our matched filter model may be applicable to recognition tasks as well. Some part of the brain would have to be organized as many little matched filters, and it would have to set the weights in a different matched filter for every picture on which the subject concentrated. Obviously, this area would need a huge capacity, but it would be much more efficient to build a large capacity memory out of synaptic weights than out of reverberating circuits. Then, during testing, the matched filter would implicitly test the incoming picture against all stored pictures simultaneously. If any little filter responded with a total power above some threshold, the familiar object would be recognized. Another advantage of this approach to recognition memory is that the recall is implicit, and thus in a sense, free. There is no computation other than the weighted sum of inputs performed by the biophysical properties of the soma. This could be vitally important to the animal, which otherwise would have to actively search through thousands of memories to find a match. No matter how large the number of matched filters, the time to test an incoming pattern is fixed regardless of memory size. In search schemes, the time would grow with memory size.

### Synaptic Versus Reverberating Memory Trace

Delay period activity in prefrontal cortex during memory tasks, which is proposed as playing a critical role in storing sensory signals, has been an important discovery for unraveling neuronal mechanisms of working memory since the 1970s [21],[54],[55]. However, delay period activity in prefrontal cortex is related to storing the sensory signal, and that signal can be modulated depending on whether the stored signal would be used to execute or suppress an action, that is, for response selection, indicating that the delay activity is also, or even mainly, related to executive function [26],[56]. A recent study shows that prefrontal cortex holds the decision during a memory task, while the middle temporal visual area computes comparisons between sample and test visual motion [56]. Our proposal supports the suggestion that stimulus-selective working memory signals are held in higher sensory areas (area TE in our case, MT for Zaksas and Pasternak [56]).

### Theory of Working Memory

Our study shows evidence for working memory storage with a silent storage mechanism using rapidly adapting synaptic weights, and a matched filter provides a specific proposal of how to utilize the outputs of TE neurons to detect a match. Our new model explains the noise correlations across time in our data. The matched filter theory also formulates and answers many important questions about the mechanism of visual working memory: what is remembered (the entire output of the encoder population); how and where it is remembered (as synaptic weights of TE neurons); how it is recalled (recall is not needed in a matched filter mechanism); and how the memory trace is compared with a new response (correlation by multiplication of input activity and synaptic weights). The output of TE could thus be used to make the match/nonmatch decision, simply by applying a threshold to the total population activity.

### Conclusion

Our hypothesis arose from the need to explain the noise correlation seen in our experimental data from area TE of inferior temporal cortex. If the correlations in our data were due to an artifact arising from some stimulus-dependence that remained after the mean was subtracted, we might have seen a similar pattern of correlations in perirhinal cortex. Also, breaking the serial relationships within single trials by shuffling the match responses within each stimulus pattern group should not have destroyed the correlations (cf. Results). If the correlations arose from a slowly varying signal having nothing to do with memory or the stimulus (e.g., due to arousal) the correlations should be higher between sample and nonmatch than between sample and match in sample-nonmatch-match trials. Thus, our explanation for the correlations between noise in sample and test responses in TE neurons is that they are a consequence of the multiplicative mechanism in a local, synaptic, short-term memory.

This model does not specify how a neuronal circuit might perform matched-filtering. Cortical architecture, connections and dynamics are all ignored. Nonetheless, this model does more than merely provide a representation of the data. Our data show only that cells are selective for stimuli, respond differently depending upon the condition (sample, nonmatch, match), and that there is correlated noise on their responses across sample-test presentations. The filter model demonstrates (thus, it is an existence proof) that the responses themselves (not the noise) could come from a multiplicative interaction between a synaptic memory trace and a current input. Note that this part of the model is parameter-free: there is no tuning or fitting involved. Thus, it is a very strong argument in favor of the matched filter theory of short-term memory, even without a biophysically detailed model. Furthermore, the matched filter hypothesis explains why the noise correlation exists, even though it makes no contribution to solving the memory task. It is important to remember that the noise-correlations are not built into the model by fitting it to the data (e.g, perirhinal neurons are fit the same way but have no such correlations). Thus, this “black-box” filter model, although simplistic with respect to cortical circuitry, provides a mechanistic explanation of how the noise correlations could arise, and thus is a plausible model for short-term memory.

## Methods

Two monkeys (*Macaca mulatta*) were trained to perform a sequential delayed match-to-sample (DMS) task. The monkeys squatted in a primate chair 57 cm in front of a rear projection screen (90° visual angle) with a black and white random dot pattern. Trials began when the monkey touched a bar on the chair. A fixation point (0.5°×0.5°) appeared and the monkey was required to fixate loosely (within ±5° of the fixation spot) for the whole trial. When patterns used in the DMS task (8.5°×8.5°, Figure 1B) appeared, it obscured the fixation point (see Figure 1A for timings). Because we were studying working memory, we used a set of very familiar visual patterns, ones that the monkeys had seen thousands of times each, to avoid any effects due to novelty. Over ninety percent of the trials were divided between trials with no nonmatches or one nonmatch. The other trials had two or more nonmatch stimuli to make it difficult for the monkeys to anticipate the match stimulus (the monkeys generally performed these trials correctly). Because the numbers of trials with 2 or more nonmatching trials were small (typically 1–4%), data were analyzed only from the trials with either zero or one nonmatch stimulus. An error was registered either when the monkey did not release the touch bar during the two seconds after the original stimulus reappeared (matching stimulus) or when the monkey moved its eyes beyond the fixation limit. Only data from correct trials were analyzed. The mean reaction times for these two monkeys were ≤500 ms.

Tungsten microelectrodes (Roboz-Microprobe, Rockville, MD) were used to record single units. Principal components were used to select well-isolated single units [57]. TE recording was carried out in the area from +14 to +17 on the anterior-posterior plane lateral to the anterior medial temporal sulcus, and perirhinal recording was done from +17 to +23 on the anterior-posterior plane medial to the anterior medial temporal sulcus as shown in Figure 12 [13]. To confirm the recording locations were within area TE, MR images were obtained with tungsten electrodes still in place (shown schematically in Figure 12B) after some of the recording sessions [13]. All of the experimental procedures were conducted in accordance with the NIH Guide for the Care and Use of Laboratory Animals and were approved by the Animal Care and Use Committee of the NIMH.

Neuronal responses were quantified by the number of spikes occurring between 70 and 470 ms after appearance of the sample, nonmatch, and match stimulus for TE neurons, and between 120 and 520 ms for perirhinal cortex neurons.

All data analyses were done in the R statistical computing environment [58]. We calculated the linear regressions between responses across different task phases to quantify the variance in the correlations across time epochs. Correlations were also done with parametric (Pearson) measures [59]. To investigate whether any violations of classical regression assumptions affected our results, we repeated our analyses using robust regression methods [58]. Our results were essentially unchanged, so we present results from standard linear regression. A square-root transformation of spike count to reduce heterogeneity of response variance [60] did not change our results, so we report the results from the untransformed data.

### Simulations

Computer simulations of the matched filter model of TE were carried out in MATLAB (The MathWorks, Natick, MA). The scalar matched filter model used for the data analysis has no parameters. To introduce a stochastic vector model of physiological neuronal data with noise, three kinds of noise were added to the model: slowly varying input noise common to all neurons (δ), encoder noise (α_km_) and population noise (β_km_) (Figure 4). Because the variance of a neuronal response is often proportional to its amplitude, we used multiplicative encoder noise. The other noise sources were additive. (An important point to make here is that we chose the matched filter model because it is the simplest nonlinear model that can explain our results, not because it is the only model that can do so.) The three noise parameters were fit to the data using the simplex algorithm in the *modeFrontier* optimization program (ESTECO, Italy).

The input to the model is an encoding of the image by a population of visual system neurons. Because the exact nature of the visual input signals are not known for either area TE or perirhinal cortex, we chose an arbitrary encoding, where each of 256 neurons represented one component of a two-dimensional, 16×16 pixel, discrete Fourier transform (DFT). These 256 neurons create a visual encoding of 8×8 Walsh images on a 16×16 gray background (e.g., Figure 8). The 256 encoder neurons projected to 256 inputs of a matched filter.

On each trial, for each neuron, for each pattern, a new sample of white noise was chosen, uniformly distributed in the range 1+(−K_α_, K_α_). The value of K_α_ sets the range of the simulated data. Population noise was drawn from a white noise process with a normal distribution (mean zero and standard deviation K_β_). When K_β_ was zero, the matched filter discriminated the match stimulus perfectly. As K_β_ increased discrimination decreased. For each neuron, there is a draw of alpha and beta from the same distribution for each stimulus. A shared input noise, δ, the same for all neurons and all phases (sample, nonmatch, and match) in a single trial, was used to test the hypothesis that the noise correlations between sample and match came from exogenous sources. The δ noise was common to all the neurons, as might happen if the animal's attention or level of arousal changed from trial-to-trial. The result was to change the values of noise correlation of the sample response with the nonmatch and match response together (not shown), whereas the data showed a higher correlation between noise on sample and match than on sample and nonmatch.

DMS trials were repeated 40 times to match the size of the experimental data sets for each neuron. This process was repeated 30 times to match the number of neurons. Images are shown with a logarithmic gray scale. Model responses are plotted as the normalized sum of the squared magnitude of the discrete Fourier transform. Frequency-domain figures contain data with a wide range of values. To better visualize the low values (where the noise is most apparent), we plotted the logarithmically transformed values of each cell, z, using *log(1+z)*.

Although the matched filter model is parameter-free, the stochastic model needed three parameters to simulate the type and amount of noise in the neuronal responses. Noise parameters of the model were adjusted to fit the mean values of the data in Figure 10, and to perform the DMS task successfully (high power for match, low power for nonmatch stimuli). The simplex does not converge to a fixed point for this model, because each run contains a new noise sample. Instead of a fixed point the parameters approach a limit cycle. The values used to obtain the data summarized in Figure 10 were: K_α_ = 0.482, K_β = _0.094, and δ = 0.01 (TE) or 0.097 (perirhinal).
